# Interleukin‐6 as a Potential Biomarker for Alzheimer’s Disease: A Meta‐Analysis with Moderator Effects

**DOI:** 10.1002/alz70861_108694

**Published:** 2025-12-23

**Authors:** Jin Mi Chun, Ji‐Woo Suk, Kahye Kim, Jaeuk Kim

**Affiliations:** ^1^ Korea Institute of Oriental Medicine, Daejeon, Yuseong Gu Korea, Republic of (South); ^2^ Korea Institute of Oriental Medicine, Daejeon, Daejeon Korea, Republic of (South)

## Abstract

**Background:**

Alzheimer’s disease (AD) is a chronic, progressive neurodegenerative disorder primarily characterized by cognitive decline, memory loss, and functional impairment. Although current diagnostic practices rely heavily on neuroimaging and neuropsychological assessments, these approaches are often expensive, time‐consuming, and limited in accessibility. Consequently, there is a growing demand for noninvasive, cost‐effective peripheral biomarkers that can facilitate early diagnosis and monitor disease progression. Interleukin‐6 (IL‐6), a pro‐inflammatory cytokine involved in neuroinflammation and microglial activation, has been proposed as a potential biomarker. Previous studies have reported elevated IL‐6 levels in both blood and cerebrospinal fluid (CSF) of AD patients. However, the literature presents inconsistent findings regarding IL‐6 dynamics across AD stages, with some studies suggesting an increase with disease progression and others reporting no change or even a decrease. These discrepancies may result from differences in study design, sample size, population characteristics, or measurement methods.

**Methods:**

To clarify these inconsistencies, we conducted a comprehensive meta‐analysis and moderator analysis. Twenty‐two studies published up to 2024 were included, all comparing IL‐6 levels between AD patients and cognitively healthy controls. Data were collected from international databases including PubMed, Embase, and Medline. A random‐effects model using Restricted Maximum Likelihood (REML) estimation was applied. Moderator variables included AD severity (mild, moderate, severe) and the biological source of IL‐6 measurements (serum, plasma, or CSF). Publication bias was assessed using Egger’s test and evaluation of funnel plot asymmetry.

**Results:**

The meta‐analysis demonstrated that IL‐6 levels were significantly higher in AD patients than in controls (SMD [95% CI] = 1.431 [0.839, 2.023]). Significant heterogeneity was noted (I² = 96.87%). Moderator analysis revealed a progressive increase in IL‐6 levels corresponding to AD severity: mild (SMD = 0.88 [0.21, 1.55]), moderate (1.39 [0.62, 2.15]), and severe (2.58 [1.10, 4.06]). Additionally, IL‐6 concentrations were markedly lower in CSF compared to serum, suggesting the influence of sample matrix on detectability.

**Conclusion:**

These findings support IL‐6 as a potential peripheral biomarker for both diagnosis and disease severity assessment in AD. However, standardized protocols and larger prospective studies are essential to validate its clinical applicability and improve reproducibility.

